# Lactic Acid-Based Natural Deep Eutectic Solvents to Extract Bioactives from Marine By-Products

**DOI:** 10.3390/molecules27144356

**Published:** 2022-07-07

**Authors:** Maha M. Abdallah, Martim Cardeira, Ana A. Matias, Maria Rosário Bronze, Naiara Fernández

**Affiliations:** 1 iBET, Instituto de Biologia Experimental e Tecnológica, Apartado 12, 2781-901 Oeiras, Portugal; maha.abdallah@ibet.pt (M.M.A.); martim.cardeira@ibet.pt (M.C.); amatias@ibet.pt (A.A.M.); mbronze@ibet.pt (M.R.B.); 2 Instituto de Tecnologia Química e Biológica António Xavier, Universidade Nova de Lisboa, Av. da República, 2780-157 Oeiras, Portugal; 3 Faculdade de Farmácia, Universidade de Lisboa, Av. Prof. Gama Pinto, 1649-003 Lisboa, Portugal

**Keywords:** natural deep eutectic solvents, extraction process, marine waste valorization, physicochemical characterization, biocompatibility

## Abstract

Natural deep eutectic solvents (NaDES) were used to extract bioactive compounds from marine by-products: codfish bones, mussel meat, and tuna vitreous humor. NaDES were prepared using natural compounds, including lactic acid (Lac), fructose (Fru), and urea (Ur), and were characterized to define their physicochemical properties, including the viscosity, density, surface tension, and refractive index. FTIR and NMR analysis confirmed the presence of intermolecular hydrogen bonding in NaDES. The extracts obtained using these NaDES were characterized to define their composition. Results demonstrated that the extract’s composition differed highly, depending not only on the DES used, but also on the structure and composition of the raw material. Proteins and lipids were mainly present in extracts obtained from mussels, while ash content was highest in the extracts obtained from codfish bones. The biocompatibility of NaDES and the soluble fractions (SF) of the raw materials in NaDES was evaluated, and it was possible to conclude that the soluble ingredients obtained from the raw materials improved the biocompatibility of NaDES.

## 1. Introduction

The total mass of marine-generated by-products was estimated to be approximately 20 million tons globally [1]. These wastes are generally discarded on land or in the sea and can lead to the contamination of the coastal water [2]. Marine by-products have been largely investigated for their valorization and use in industrial applications due to their abundance, low-cost, and environmental advantages [3]. Therefore, their use as a source for the isolation of bioactive compounds has great advantages as nowadays the integration of natural products in pharmaceutical and cosmetic industries has remarkably increased, as their biological activity and structural properties may differ from the synthetic molecules [4,5]. Many compounds used in pharmaceutical applications were originally isolated from natural sources, such as plants and biomass [6]. For example, vitamin C was obtained from citrus, salicylic acid from willow bark, taxol from yew bark, pilocarpine from Pilocarpus microphyllus leaves, and quinine from cinchona bark, among many others [7]. Natural proteins obtained from plant and animal sources have been used in drug and gene delivery systems, including hydrogels, nanocarriers, and nanoparticles [8,9]. Moreover, natural lipids were also used in cosmetics, pharmaceuticals, and nutritional supplements. They include oils obtained from plants and animals, natural fats, waxes, and phospholipids [10]. For instance, natural retinoids, carotenoids, and tocopherols were employed in medicinal applications due to their antioxidant properties [11,12,13]. Furthermore, marine by-products were highly employed as a source of natural ingredients due to their potent and bioactive properties [14]. Hence, a considerable amount of the inedible parts of fish by-products is discarded globally from processing industries, which includes skin, trimmings, heads, fins, and viscera [1,15]. Marine by-products are mainly used as a source of several bioactive compounds, such as proteins (collagen and gelatin), fatty acids, minerals (calcium phosphate and hydroxyapatite), and enzymes (pepsin, collagenases, trypsin, and chymotrypsin) [14].

Natural deep eutectic solvents (NaDES) were widely employed as alternative solvents to isolate bioactive compounds [16]. NaDES are green solvents used in several applications as they present promising advantageous characteristics due to their low-cost, low- to non-toxicity, and biodegradability for environmental-friendly processes [17,18,19]. They are defined as novel eutectic mixtures having a lower melting point than the pure substances used to prepare them, which is caused by the complexation of the hydrogen bond acceptor and hydrogen bond donor molecules in the system [20]. They have been widely investigated as analogues to ionic liquids and were proven to be more eco-friendly and cheaper alternatives to ionic liquids and other conventional solvents [21,22]. Some limitations of ionic liquids in natural products research are their negligible volatility and high viscosity [23,24]. NaDES include solvents with lower viscosity and more favorable, sustainable, and biocompatible properties, which was shown to be advantageous for their use in health-related applications [25]. They present a wide combination of compounds for their synthesis at a diverse tunability [17,26]. This has allowed the optimization of the processes that employs these solvents to enhance the yield, solubility, and selectivity, among different physicochemical properties of interest, depending on the application [27,28]. Previous studies have shown the potential uses of versatile NaDES in various applications [18], including the pre-treatment, purification, extraction, recovery, and synthesis of materials from natural sources [29,30,31]. For instance, they demonstrated to be potential novel solvents in the extraction of bioactive compounds, including proteins, carbohydrate polymers, phenolic compounds, flavonoids, and fatty acids, among others [32,33,34,35]. In this work, the selected by-products as a source of bioactive molecules were codfish bones (CB), mussel meat (MM), and tuna vitreous humor (TVH). These materials are abundantly discarded in fishery processing industries, either because they are not edible, or not safe for human consumption [36,37,38]. Hence, various methods were developed for the efficient extraction of valuable ingredients from biomass sources [39]. Lactic acid-based NaDES were synthesized in order to be used for the isolation of extracts from marine raw material as a potential technique to obtain bioactive natural compounds, including proteins, lipids, and minerals. The extraction using these novel solvents is compared to the raw material composition using conventional methods for the extraction of the mentioned compounds, to evaluate their viability as an alternative extraction approach.

## 2. Results and Discussion

### 2.1. Extraction Process

NaDES were used for the extraction of bioactive ingredients from natural marine by-products that are abundantly discarded. This method could not only ensure the isolation of natural compounds, but also implement the valorization of discarded marine wastes by employing a green and low-cost process using NaDES.

**Figure 1:**
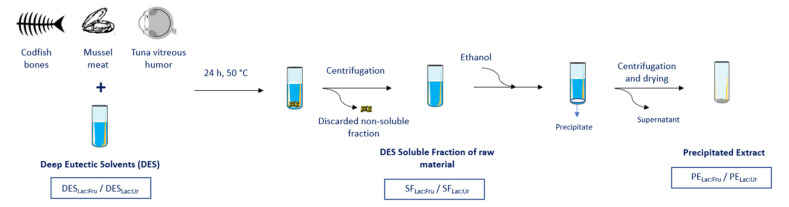
Scheme of the extraction process.

NaDES were prepared using lactic acid; fructose; and urea as low-cost, natural, and non-toxic molecules [40,41,42]. Taking into consideration the applicability and relevance of these compounds, two combinations of NaDES were synthesized by mixing lactic acid (Lac) with either fructose (Fru) or urea (Ur) (Lac:Fru and Lac:Ur, respectively). Figure 1 shows the scheme of the extraction process that is used in this work. After mixing NaDES with the marine raw materials, the undissolved part of by-products was discarded and the raw material’s soluble fraction in NaDES was obtained (SF_Lac:Fru_ and SF_Lac:Ur_). The samples of the process were characterized and tested in order to evaluate their biocompatibility. This would allow the use of NaDES not only as an extracting green solvent, but also as part of a therapeutic system containing the marine by-products soluble fraction.

### 2.2. NaDES Characterization

#### 2.2.1. FTIR Analysis

**Figure 2:**
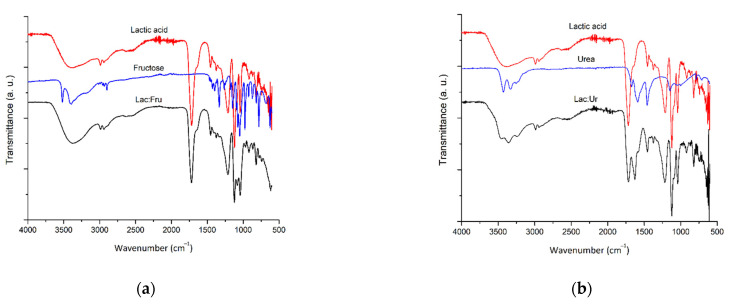
FTIR spectra of the pure compounds and the synthesized natural deep eutectic solvents (**a**) Lactic acid:Fructose (Lac:Fru, 5:1), and (**b**) Lactic acid:Urea (Lac:Ur, 4:1).

Figure 2 displays the FTIR of the pure components and the solvent systems synthesized at the eutectic molar ratio. The spectra of the prepared solvents exhibited similar absorption profiles to those of the pure components, with lactic acid having the dominant vibrating peaks in solvent systems as it has a higher molar ratio (5:1 and 4:1 of Lac:Fru and Lac:Ur, respectively). A strong absorption band was observed in lactic acid and the solvents at 1718 cm^−1^, which was a relative to the C=O stretching vibration present in the lactic acid. In contrast, the broad absorption peak at 3361 cm^−1^ corresponded to the O–H bond in a carboxylic acid of the pure lactic acid and in the synthesized solvents. This stretching bond was also visible in the spectra of fructose at 3394 cm^−1^. It can be observed in both systems Lac:Ur and Lac:Fru that the wavenumber of the O−H stretching peaks shifted in comparison to the pure compounds as hydrogen bonding occurred. Moreover, the bands 1050 and 976 cm^−1^ were relative to the strong absorption of the C–O stretching bands. For urea, the bands at 1460, 1589, and 1672 cm^−1^ represent the C–N, N–H, and C=O stretching vibrations, respectively [17,43]. The N–H and C=O peaks were shifted to higher wavenumbers in Lac:Ur to 1628 and 1714 cm^−1^, respectively, due to hydrogen bonding that took place.

#### 2.2.2. NMR Analysis

**Figure 3:**
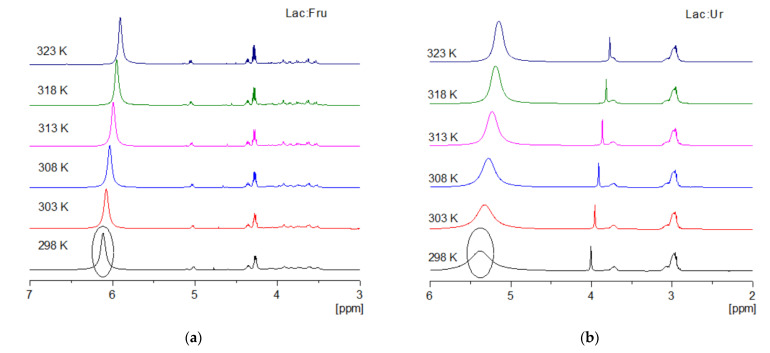
^1^H-NMR spectra of natural deep eutectic solvents: (**a**) Lactic acid:Fructose (Lac:Fru) and (**b**) Lactic acid:Urea (Lac:Ur) at different temperatures.

**Figure 4:**
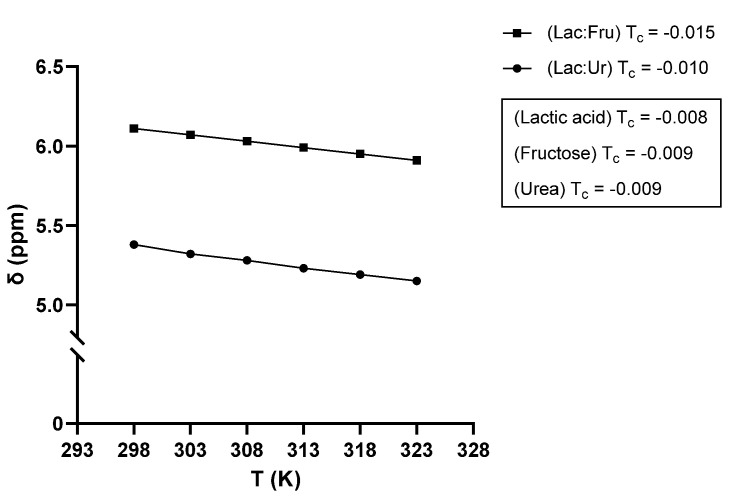
Plot of the chemical shifts (δ) of the OH protons as a function of temperature.

The ^1^H-NMR spectra of Lac:Fru and Lac:Ur is shown in Figure 3. These systems were studied at different temperatures from 25 to 50 °C to evaluate the behavior of the H-bonded protons for the inter- and intra-molecular bonds [44]. For Lac:Fr, a chemical shift of the –OH protons occurred from 6.11 to 5.91 ppm when increasing the temperature, as shown in the circled peak of Figure 3a. Hence, as the temperature increases, the H-bonded protons undergo an upfield shift [44,45,46,47]. A similar behavior was observed for Lac:Ur, in which a broad peak was observed at 5.38 ppm and it shifted to a lower wavelength while becoming less broad with the temperature increase until it reached 5.15 ppm at 50 °C, as shown in the circled peak of Figure 3b. In order to evaluate the magnitude and the type of H-bonding chemical shift, a temperature coefficient (T_c_) is calculated, as shown in Figure 4. It was obtained from the slope of the linear correlation of the chemical shifts (δ) as a function of the system temperature. When T_c_ is more negative than −0.005 ppm/°C, an intermolecular H-bonding occurs, while when T_c_ is less negative than −0.003 ppm/°C, an intramolecular H-bonding occurs [44]. The T_c_ values of both chemical shifts in Lac:Fru and Lac:Ur are shown to be more negative than −0.005 ppm/°C, suggesting that an intermolecular H-bonding occurred in the system. The chemical shift was also studied for the pure lactic acid, urea, and fructose. Results show that an upfield shift of the –OH functional group is also observed when the temperature is increased. However, the extent of the chemical shift is lower than that observed in the eutectic systems (Figure 4). Consequently, the molecular interaction differs between the pure compounds and the eutectic systems, suggesting that hydrogen bonding occurs between the different molecules of the eutectic systems and that they do not act as an ideal mixture and behave as NaDES.

#### 2.2.3. Physical Properties

In-depth analysis of the various physicochemical properties of DES is required for their use as novel and alternative sustainable solvents in industrial fields, in order to understand the molecular interactions and dynamics and to define the thermodynamic behavior. The viscosity is an important parameter that should be assessed for the application of the DES in the extraction and treatment of a target material. Many DES exhibit high viscosities at room temperature, which is less favored for their application as extractants. The high viscosity is mainly due to the extensive hydrogen bonding, which decreases the mobility of free species in the system [17,48]. This parameter also depends on the chemical structure of the components in the system. For instance, it was shown to be higher with the increase of the tetraalkylquaternary ammonium chains and to decrease with the presence of long-branched chains [48]. For the NaDES used in this study, the viscosity was shown to be 746.70 and 732.91 mPa/s for Lac:Fru and Lac:Ur, respectively, at room temperature (25 °C), as shown in Table 1. At the extraction temperature of 50 °C, this value decreased to 113.40 and 187.93 mPa/s for Lac:Fru and Lac:Ur, respectively, which would be more favorable in the extraction process. The viscosities of these lactic acid-based NaDES were shown to be comparable to those of choline chloride:urea (1:2) system, which were shown to be 748.09 and 119.81 mPa/s at 25 and 50 °C, respectively [49]. In contrast, the lactic acid-based NaDES were shown to be much more viscous than terpene-based NaDES, as the values were 19.23 and 45.63 mPa/s for menthol:camphor (3:2) and thymol:borneol (7:3) at 25 °C, respectively [47]. Hence, this proved that this property highly differs in the chemical composition and structure in the systems.

Another important parameter for the application of DES in industrial applications is the density, which also depends on the structure of the components of the system [49,50]. It is essential to determine this parameter in extraction processes as it affects the kinetic rate and the driving force between the solvent and the solid particles [48,51]. The density of the lactic acid-based NaDES was shown to be 1.2 g/mL (Table 1). Many studies were conducted to evaluate the density of deep eutectic solvents [27,52,53]. For instance, the density of choline chloride:glycerol and choline chloride:ethylene glycol (1:2 of molar mass each) is shown to be 1.18 and 1.12 g/mL, respectively; while ZnCl_2_-based eutectic mixtures exhibit a higher density with a value of 1.36 and 1.63 g/mL, for ZnCl_2_:acetamide (1:4 in molar mass) and ZnCl_2_:urea (1:3.5 in molar mass), respectively [17,48,54]. The difference in the densities between each combination of DES is affected by the molecular organization and packing within the system [17].

Furthermore, the surface tension and refractive index of solvents play an essential role in the design of various unit operations for the industrial application. They highly depend on the level of intermolecular interaction of the molecules [55,56]. The surface tension helps to understand the behavior of solvents applied in processes and the cohesive forces between the molecules within the solvent [57]. The values were shown to be 44.62 and 43.96 mN/m for Lac:Fru and Lac:Ur, respectively (Table 1). These values are comparable to ones found in the literature as the surface tensions of choline chloride:phenylacetic acid (1:2 in molar mass) and 1-butyl-3-methyl-imidazolium tetrafluoroborate have been reported to be 41.86 and 44.81 mN/m, respectively [21,58]. As for the refractive index, the values were shown to be 1.46 and 1.44 for Lac:Fru and Lac:Ur, respectively, as shown in Table 1. Previous studies demonstrated comparable results as the values for tetrabutylammonium bromide:3-amino-1-propanol (1:4 of molar ratio) and tetraethylammonium chloride:3-amino-1-propanol (1:8 of molar ratio) were 1.4807 and 1.4717, respectively [53].

### 2.3. Extracts Characterization

The extraction yield using each NaDES is shown in Table 2. Based on these results, it can be concluded that the extraction mass yield of the extracts ranges from 9.3 to 22.5% (*w*/*w*). The yield was the highest for extracts obtained from MM in comparison to the other raw materials. The chemical composition in lipids, proteins, and ash per 100 mg extract is displayed in Table 3 and their yield per 100 mg raw material is shown in Table S1.

According to the results, proteins present the highest content in the obtained extracts. For CB, proteins percentage is 53.12 and 57.63% of the extracts using Lac:Fru and Lac:Ur, respectively. This concentration corresponds to 5.07 and 6.70% of codfish bones using Lac:Fru and Lac:Ur, respectively (Table S1). A much higher ash content was determined from this raw material in comparison to lipids. According to the results, Lac:Ur was shown to be most suitable for the extraction of proteins from CB, as it was able to extract around 1/6 of the total proteins from this raw material. Furthermore, when comparing the extracts content with the raw material composition (Table S1), it can be concluded that the bones contain mainly ash (54.35%), followed by proteins (38.59%). Previous studies demonstrated that 81.09–84.88% of proteins were present in fish bones [59], and 39.43 and 58.01% of ash and proteins, respectively, in codfish bones [60]. These results vary depending on different factors that can contribute to the variation in chemical composition of these raw materials, such as the local of capture, season, and nutrient intake, among others. In addition, it is generally possible to find leftovers of meat and proteins with the bones, as when the meat is separated for commercial use, there could be some meat that remains with the discarded bones.

In the extracts obtained from MM, a higher percentage of proteins was extracted in comparison to the other raw materials, with a value of 66.11 and 75.20% of protein in the extracts, obtained using Lac:Fru and Lac:Ur, respectively. The ash content in these extracts is slightly higher than the lipid content using both NaDES. The characterization of this raw material showed that it was mainly composed of proteins (51.32%), followed by ash (17.57%) and lipids (15.41%), as shown in Table S1. As previously discussed, also the chemical composition of this raw material is highly variable based on seasonal and geographical factors [61]. For this raw material, Lac:Ur was also the more suitable NaDES to extract the highest yield of protein, as it isolated around 1/3 of the total protein present in MM.

The extracts obtained from TVH were composed of 50% of proteins in the extracts using both NaDES, equivalent to 5.16 and 7.43% of proteins of the raw material using Lac:Fru and Lac:Ur, respectively. Hence, these DES were able to extract around half of the total proteins present in TVH. Consequently, Lac:Ur had the highest capacity to extract proteins from all raw materials used. In addition, the ash content was shown to be 27.98 and 29.03% of the extracts using Lac:Fru and Lac:Ur, respectively, which are higher than the amount of lipids isolated from these extracts. The analysis of the TVH composition showed that it was mainly composed of ash (62.26%), followed by proteins (14.31%) and lipids (8.06%). By comparing these results to previous studies, it can be concluded that the composition varied as the amounts were 50.78, 30.89, and 7.32% of ash, protein, and lipids, respectively [62,63]. Hence, this biomass material is also highly variant in composition depending on its source. For all raw materials, the extracts isolated using both DES contained other remaining compounds, which could include additional carbohydrate polymers and polysaccharides isolated using DES [19,31,32].

As a conclusion, the lactic acid-based NaDES efficiently extracted bioactive compounds at varying compositions, depending on the raw material and NaDES used, and their bioactivity was further analyzed to confirm their safety in potential therapeutic applications.

### 2.4. Biocompatibility

The biocompatibility studies of NaDES and SF testing samples were evaluated and compared to study the potential therapeutic effect of the samples. The study was done in the Caco-2 cell line as it has been shown to be considered an accepted model for biocompatibility evaluation [64,65,66]. Previous studies showed that the undifferentiated Caco-2 cell line has a higher sensitivity to the compounds that were tested in comparison to differentiated cells. Hence, this line can be considered as one of the most conservative models for the characterization of hazards [66].

**Figure 5:**
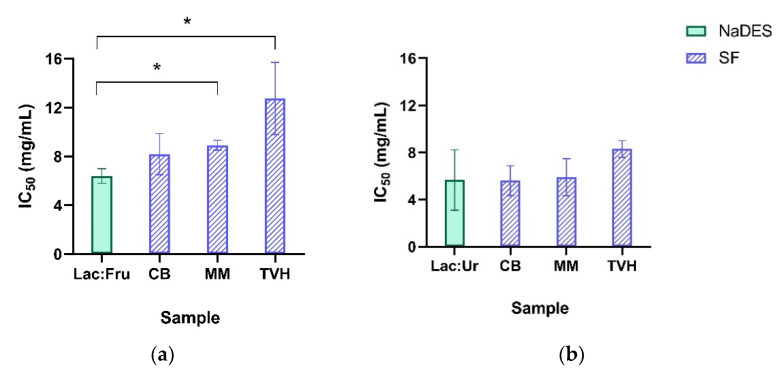
Half inhibitory concentration (IC_50_) values of the natural deep eutectic solvents (NaDES) samples: (**a**) Lactic acid:Fructose (Lac:Fru) and (**b**) Lactic acid:Urea (Lac:Ur), and the soluble fraction (SF) in each NaDES of the raw materials codfish bones (CB), mussel meat (MM), and tuna vitreous humor (TVH). Statistical significance * *p* ≤ 0.05 (*n* = 3).

The half inhibitory concentration (IC_50_) of the NaDES was compared to that of SF samples, as shown in Figure 5. Results showed that the IC_50_ value of Lac:Fru (6.39 mg/mL) was significantly lower than that of the SF_Lac:Fru_ for MM and TVH (8.92 and 12.74 mg/mL, respectively). Therefore, Lac:Fru was shown to be significantly less biocompatible than SF_Lac:Fru_ due to higher cytotoxicity (lower IC_50_ values). As for Lac:Ur, the IC_50_ value (5.68 mg/mL) was comparable to that of the SF_Lac:Ur_ of CB and MM (5.61 and 5.90 mg/mL, respectively) and lower than that of SF_Lac:Ur_ of TVH (8.30 mg/mL). Hence, SF_Lac:Ur_ of these raw materials were viable to the cells but did not show any significant increase of the biocompatibility of Lac:Ur. Therefore, the cellular viability differed based on the NaDES used, with Lac:Fru samples being significantly more viable to the cells and on the raw material from which the soluble fraction was dissolved, with TVH being the most viable.

## 3. Materials and Methods

### 3.1. Materials

Codfish bones were kindly donated from Pascoal & Filhos (Nazaré, Portugal), mussels from Testa & Cunhas (Nazaré, Portugal), and tuna eyes from Tunipex (Faro, Portugal). Tuna eyes were kept frozen until cut, to separate the vitreous humor from the eyeball.

The reagents used in NaDES and extracts characterization were the following: DL-lactic acid (aqueous solution, 85.0–90.0%, CAS: 50-21-5) was obtained from Alfa Aesar, Haverhill, MA, USA. D-fructose (CAS: 57-48-7), urea (CAS: 57-13-6), 2,2-Dimethyl-2-silapentane-5-sulfonate (DSS, CAS: 2039-96-5), deuterium oxide (D2O, CAS: 7789-20-0), chloroform (CAS: 67-66-3), hydrochloric acid (≥37%, CAS: 7647-01-0), sodium chloride (CAS: 7647-14-5), disodium hydrogen phosphate (CAS: 7558-79-4), sodium tetraborate (99%, CAS: 1330-43-4), Trizma^®^ base (≥99.9%, CAS: 77-86-1), glycine (≥99%, CAS: 56-40-6), sodium dodecyl sulfate (≥99%, CAS: 151-121-3), and Folin & Ciocalteu’s Phenol Reagent (2N, CAS: 12111-13-6) were obtained from Sigma-Aldrich, Waltham, MA, USA. Methanol (≥99.8%, CAS: 67-56-1) from Carlo Erba Reagents, Val de Reuil, France. Sodium phosphate (96%, CAS: 7601-54-9) from Aldrich, Waltham, MA, USA. Sodium hydroxide (98%, pellets, CAS: 1310-73-2) from Acros Organics, Geel, Belgium. The reagents used to prepare the complex-forming reagent of Lowry’s method are the following: sodium carbonate (anhydrous, CAS: 497-19-8) from Panreac, Barcelona, Spain; Copper(II) sulfate pentahydrate (> 99%, CAS: 7758-99-8) from Acros Organics, Geel, Belgium; Potassium sodium tartrate tetrahydrate (≥99%, CAS: 6381-59-5); and bovine serum albumin (≥96%, CAS: 9048-46-8) from Sigma-Aldrich, Waltham, MA, USA.

The reagents used for the in-vitro studies were the following: Dulbecco’s Modified Eagle Medium (DMEM), non-essential amino acids (NEAA), and penicillin-streptomycin (PenStrep) were purchased from Invitrogen (Gibco, Paisley, UK). Heat-inactivated fetal bovine serum (FBS) was obtained from Biowest (Riverside, MO, USA). Phosphate buffered saline (PBS) was purchased from (Sigma-Aldrich, St. Louis, MO, USA). PrestoBlue^®^ Cell Viability Reagent (Molecular Probes^®^) was obtained from Life Technologies (Carlsbad, OR, USA).

### 3.2. NaDES Synthesis and Characterization

NaDES were synthesized by mixing and heating (at 80 °C) the compounds at a specific molar ratio [67,68] (Table 4) for 15 min to ensure the formation of a homogenous liquid.

The analysis of the structure and interactions in these solvents, Fourier-transform infrared spectroscopy (FTIR) (Class 1 Laser Product Nicolet 6100, San Jose, CA, USA) and Nuclear Magnetic Resonance (NMR) (Avance II 500 spectrometer, Bruker, Rheinstetten, Germany) were used. For FTIR, the absorption spectra were recorded in the range of 4000–600 cm^−1^ with 4 cm^−1^ resolution and 40 scans of the samples. In addition, ^1^H NMR spectra was recorded at 500 MHz at 30° pulse angle, 4.5-s pulse delay, and 16 scans. 2,2-Dimethyl-2-silapentane-5-sulfonate (DSS) and deuterium oxide (D2O) were placed in a capillary tube inside the NMR tube containing 300 µL of the solvent systems. The sample was locked using the frequency of the DSS. At first, the sample was equilibrated using a thermocouple-T for 10 min to monitor the temperature and adjust it to 25, 30, 35, 40, 45, and 50 °C. Proton chemical shifts were calibrated in reference to DSS.

The viscosity of NaDES was studied using a rheometer (MCR 102, Anton Paar). The equipment was fitted with a parallel plate geometry (PP50-61752) with a gap of 0.8 mm and a constant shear rate of 10 s^−1^ was used. At first, the samples were pre-equilibrated at 50 °C, then a temperature scan was done from 50 to 20 °C at a 1 °C/min cooling rate. The density was obtained by measuring the mass of 1 mL of NaDES at 25 °C. The surface tension was obtained using KSV Attension tensiometer (KSV Sigma 702), using a platinum–iridium Du Noüy ring method for surface and interfacial measurements. The ring height, diameter, and wire thickness are 25, 18.7, and 0.37 mm, respectively. The measurements were done in a thermostat bath (Lab Companion RM0525G). For the refractive index, Abbe refractometer was employed using natural light. All runs were carried out in triplicate.

### 3.3. Extraction Process

All three raw materials were freeze-dried, and codfish bones were ground to a particle size less than 1 mm. Briefly, the raw materials were mixed with the NaDES at a component mass ratio of 1:100 of raw materials:NaDES. They were stirred for 24 h at 50 °C. To collect the dissolved extracts in the solvents, the mixture was first centrifuged at 6000 rpm for 15 min at 45 °C to separate the undissolved particles and collect the supernatant. These supernatants represent the samples of the soluble fractions (SF) of the raw materials in Lac:Fru (SF_Lac:Fru_) and in Lac:Ur (SF_Lac:Ur_) (Figure 1). Extract precipitation was done by the addition of 3 volumes of ethanol. The precipitate was then collected by centrifugation at 6000 rpm for 15 min at 45 °C and the samples represent the precipitated extracts (PE): PE_Lac:Fru_ and PE_Lac:Ur_.

### 3.4. Biomass Characterization

Biomass were characterized in terms of lipids, proteins, and ash content. The lipids were extracted based on the Bligh & Dyer technique [67] in order to quantify them. The proteins were extracted according to a previously described method with slight modifications [69,70]. Briefly, 1 g of freeze-dried raw material was suspended in 100 mL of ultrapure Milli-Q water. Ultra-sonication was then done for 2 h, and the sample was stirred at 4 °C overnight using a magnetic stirrer plate. The solution was then centrifuged at 4500 rpm for 30 min and the supernatant was collected and stored at 4 °C. The pellet fraction was re-suspended in 20 mL of Milli-Q water and subjected again to ultrasonication and stirring as described above. The sample was then centrifuged, and the supernatant was pooled together with the initial supernatant and mixed with 80% (*w*/*v*) of ammonium sulfate saturation, and stirred at 4 °C during 1 h. Centrifugation was done to precipitate and collect the protein fraction. Following this, the precipitates were dialyzed at 4 °C for 24 h against Milli-Q water using a 10 kDa MWCO dialysis tubing (Snake Skin™ Dialysis Tubing, Fischer Scientific, Waltham, MA, USA). The total protein content was analyzed according to the Lowry’s method [71,72]. The experiments were done in duplicate. The ash content of the samples was determined by placing them in crucibles and heating them in a high-temperature muffle furnace (Nabertherm, Lilienthal, Germany) type LT 15/13, provided with a C450 Controller. The run was conducted for 4 h at a temperature of 550 °C. The experiments were done in duplicates. The extract composition is displayed in percentage (mg compound/100 mg raw material).

### 3.5. Extracts Characterization

The total protein content in the extracts was determined according to Lowry’s method [72]. It is a biochemical calorimetric assay used to determine the total amount of protein in a solution [72]. In brief, a complex-forming reagent was prepared according to a previously reported procedure [73]. An amount of 0.2 mL of the testing samples was added to 1 mL of freshly prepared complex-forming reagent. They were then vortexed and left at room temperature for 10 min; 0.1 mL of Folin reagent (1 N) was then added and vortexed. The mixture was kept protected from light at room temperature for 30 min and the absorbance was obtained at 750 nm (GENESYS™ 10S UV-Vis Spectrophotometer, Boston, MA, USA).

The lipids and ash content were determined following the described techniques in Section 2.4.

### 3.6. Biocompatibility

The biocompatibility of the compounds was studied using non-differentiated and confluent Caco-2 cells. This cell line was employed as it displays characteristics with crypt enterocytes and has been widely implemented as a model to evaluate the effect of compounds on the intestinal function [60,74]. The cytotoxicity of the samples was analyzed to study the potential use of the compounds in future therapeutic applications. The biocompatibility of the extracts was assessed by comparing the cellular viability of NaDES with the SF samples, prior to the extract precipitation. The precipitated extracts were not evaluated due to their limited solubility in biocompatible solvents.

The assay was performed as has been previously reported with minor modifications [75]. In brief, the cells were seeded at a density of 2 × 10^4^ cells per well in a 96-well plate. The medium (DMEM + 10% FBS, 1% PenStrep and 1% NEAA) was changed every 3 days, and following 7 days of culture, the cells were confluent and were incubated for 24 h with the extracts at different concentrations diluted using the culture medium (DMEM + 0.5% FBS + 1% NEAA). In control wells, the cells were incubated with the culture medium only. After incubation, the medium was discarded, and the cellular viability was assessed using the reagent PrestoBlue^®^ following the manufacturer protocol. The fluorescence (Ex./Em. 560/590 nm) was recorded using a FL 800 microplate fluorescence reader (BioTek Instruments, Winooski, VT, USA). Cell viability was expressed as percentage of viable cells relative to the control. The half maximal inhibitory concentration (IC_50_) is a widely employed measure of the potency and efficacy of bioactive ingredients for pharmacological studies. It indicates the concentration required to inhibit cell growth by 50% of the compounds. It was assessed using the dose–response curves fit on GraphPad Prism software (Graph-Pad Software, Inc., La Jolla, CA, USA). All the experiments were done in triplicate with at least two independent assays.

## 4. Conclusions

NaDES were synthesized, characterized, and used for the extraction of bioactive compounds as a novel green technique for marine waste valorization. They were prepared using natural compounds: lactic acid, fructose, and urea. Their physicochemical characterization demonstrated the presence of hydrogen bonding and confirmed their behavior as non-ideal deep eutectic solvents. The use of NaDES has different advantages to the use of conventional extraction methods, including the elimination of enzymes and/or organic solvents and a decrease of process time and cost. The extracts obtained using these NaDES are characterized to define their composition. Results demonstrated that proteins were the main components of these extracts, mostly in extracts obtained from mussel meat. In general, lactic acid-based NaDES have a higher capacity to isolate proteins, as the extracts contained up to half of the total proteins present in all raw materials. The amounts differed based on the raw materials used, as the extraction from bones had the lowest yields due to their dense structure. Furthermore, the extracted ash and lipids amounts using both Lac:Fru and Lac:Ur were low in comparison to the amounts of ash present in the raw materials. In addition, the extracts composition highly differs, depending not only on the NaDES used, but also on the structure and composition of the raw material used. The NaDES biocompatibility was compared to that of SF for each raw material to assess the added value of the dissolved ingredients. According to the results, the biocompatibility significantly increased in the SF_Lac:Fru_ of CB and TVH, with the SF obtained from TVH leading to the highest cellular viability in NaDES. This suggests that a protective or beneficial activity was provided by the SF. Hence, the soluble bioactive ingredients improved the bioactivity of NaDES, which shows that these compounds could be further studied for their potential use in therapeutic applications, including gastrointestinal therapy. Therefore, more in-depth study must be done to guarantee the safety of the compounds. These results also showed that the precipitation step of the extracts from NaDES could be eliminated, which decreases the cost and process time of this application.

## Figures and Tables

**Table 1:** Physical properties of natural deep eutectic solvents (NaDES).

NaDES	Viscosity at 25 °C (mPa/s)	Viscosity at 50 °C (mPa/s)	Density (g/mL)	Surface Tension (mN/m)	Refractive Index
Lac:Fru	746.70 ± 16.37	113.40 ± 5.76	1.25 ± 0.23	44.62 ± 0.07	1.4590 ± 0.0004
Lac:Ur	732.91 ± 10.22	187.93 ± 9.61	1.24 ± 0.45	43.96 ± 0.03	1.4368 ± 0.0006

**Table 2:** Extraction yield % (mg extract/100 mg raw material) using lactic acid-based natural deep eutectic solvents (NaDES): Lactic acid:Fructose (Lac:Fru) and Lactic acid:Urea (Lac:Ur).

Raw Material	Lac:Fru	Lac:Ur
CB	9.3 ± 1.3	10.7 ± 2.1
MM	19.3 ± 1.8	22.5 ± 1.9
TVH	13.1 ± 1.7	15.3 ± 1.6

**Table 3:** Percent composition (mg/100 mg extract) of lipids, proteins, and ash in the extracts obtained using natural deep eutectic solvents Lactic acid:Fructose (Lac:Fru) and Lactic acid:Urea (Lac:Ur).

Raw Material	Composition	Lac:Fru	Lac:Ur
CB	Lipids	1.54	1.86
	Protein	53.12	57.63
	Ash	19.84	22.74
MM	Lipids	4.52	5.07
	Protein	66.11	75.20
	Ash	6.40	6.94
TVH	Lipids	4.97	5.71
	Protein	49.66	50.66
	Ash	27.98	29.03

**Table 4:** Molar ratio and designation of the synthesized natural deep eutectic solvents (NaDES).

NaDES	Designation	Molar Ratio
Lactic acid:Fructose	Lac:Fru	5:1
Lactic acid:Urea	Lac:Ur	4:1

## Data Availability

Not applicable.

## Supplementary Materials

The following supporting information can be downloaded at: https://www.mdpi.com/article/10.3390/molecules27144356/s1, Table S1: Percent composition (mg/100 mg raw material) of lipids, proteins, and ash extracted from each raw materials using natural deep eutectic solvents Lactic acid:Fructose (Lac:Fru), Lactic acid:Urea (Lac:Ur), and conventional extraction methods.
