# Supplementary material for: Lactic Acid-Based Natural Deep Eutectic Solvents to Extract Bioactives from Marine By-Products
Source: Molecules. 2022 Jul 7;27(14):4356. doi: 10.3390/molecules27144356 (PMC9319700; doi:10.3390/molecules27144356)
Supplement: Supplementary file 1 [file molecules-27-04356-s001.zip › molecules-1803076-supplementary.pdf]

Supplementary Material

Table S1. Percent composition (mg/ 100 mg raw material) of lipids, proteins, and ash extracted from each raw materials using natural deep eutectic solvents Lactic acid:Fructose (Lac:Fru), Lactic acid:Urea (Lac:Ur), and raw materials characterization using conventional extraction methods*.

		**Lac:Fru**	**Lac:Ur**	**Raw materials characterization**
**Codfish bones**	**Lipids**	0.14 ± 0.03	0.18 ± 0.03	2.60 ± 0.95
	**Proteins**	5.07 ± 0.02	6.70 ± 0.14	38.59 ± 4.17
	**Ash**	1.87 ± 0.04	2.63 ± 0.27	54.35 ± 0.24
**Mussel meat**	**Lipids**	0.82 ± 0.15	1.08 ± 0.03	15.41 ± 2.09
	**Proteins**	13.99 ± 0.43	18.21 ± 0.36	51.32 ± 2.78
	**Ash**	0.83 ± 0.25	1.17 ± 0.39	17.57 ± 0.50
**Tuna vitreous humor**	**Lipids**	0.52 ± 0.06	0.59 ± 0.14	8.06 ± 1.42
	**Proteins**	5.16 ± 0.15	7.43 ± 0.25	14.31 ± 1.62
	**Ash**	3.94 ± 0.22	4.77 ± 0.33	62.26 ± 0.46

* according to the methods described in section 3.4.
